# Optical Coherence Tomography Angiography Findings in Fabry Disease

**DOI:** 10.3390/jcm8040528

**Published:** 2019-04-17

**Authors:** Gilda Cennamo, Laura Giovanna Di Maio, Daniela Montorio, Fausto Tranfa, Camilla Russo, Giuseppe Pontillo, Sirio Cocozza, Roberta Esposito, Teodolinda Di Risi, Massimo Imbriaco, Letizia Spinelli, Eleonora Riccio, Antonio Pisani

**Affiliations:** 1 Eye Clinic, Public Health Department, University of Naples “Federico II”, 80100 Naples, Italy; 2 Department of Neurosciences, Reproductive Sciences and Dentistry, University of Naples “Federico II”, 80100 Naples, Italy; arual10@hotmail.it (L.G.D.M.); da.montorio@gmail.com (D.M.); fausto.tranfa@unina.it (F.T.); 3 Department of Advanced Biomedical Sciences, University of Naples “Federico II”, 80100 Naples, Italy; camilla_russo@hotmail.it (C.R.); giuseppe.pon@gmail.com (G.P.); siriococozza@hotmail.it (S.C.); robyeire@tin.it (R.E.); mimbriaco@hotmail.com (M.I.); letspine@unina.it (L.S.); 4 CEINGE-Advanced Biotechnology s.c. a. r.l., 80145 Naples, Italy; lindadirisi@gmail.com; 5 Department of Public Medicine, University of Naples “Federico II”, 80100 Naples, Italy; elyriccio@libero.it (E.R.); antonio.pisani13@gmail.com (A.P.)

**Keywords:** Fabry disease, optical coherence tomography angiography, retinal tortuosity, retinal vessel density

## Abstract

Background: Fabry disease (FD) is a X-linked recessive lysosomal storage disorder characterized by altered biodegradation of glycosphingolipids. It is a multisystem pathology, also involving ophthalmological systems that show modifications of the vessel wall due to glycosphingolipid deposits. Optical coherence tomography angiography (OCT-A) allows for an objective analysis of retinal microvasculature alterations, evaluating retinal vessel density in macular region. Methods: A total of 54 FD patients (34 females, 20 males, mean age 44.1 ± 15.6 years) and 70 controls (36 females, 34 males, mean age 42.3 ± 15.6 years) were included in this study. We evaluated vessel density in different macular areas (whole image, fovea, and parafovea) of both the superficial capillary plexus (SCP) and of the deep capillary plexus (DCP). Results: In the SCP there was a significantly lower vascular density in patients compared with controls in whole image (49.95 ± 5.17% vs. 51.99 ± 2.52%; *p <* 0.001), parafovea (52.01 ± 6.69% vs. 54.30 ± 2.61%; *p =* 0.002), and fovea (22.38 ± 9.01% vs. 29.31 ± 5.84%; *p* < 0.0001). In the DCP the vessel density was statistically increased in each macular area in patients compared with controls (54.82 ± 8.07% vs. 50.93 ± 5.46%; *p =* 0.005, 57.76 ± 7.26% vs. 53.59 ± 5.46%; *p =* 0.0001, and 39.75 ± 8.59% vs. 34.43 ± 8.68%; *p <* 0.0001 for whole image, parafovea, and fovea, respectively). Conclusion: OCT-A analysis showed that the macular vessel density was significantly reduced in the SCP and increased in the DCP in FD patients compared with controls. These findings, which might be a consequence of the alteration of vascular wall occurring in FD, support the hypothesis that the evaluation of early retinal microvascular network changes could be a useful tool in the clinical evaluation of the disease.

## 1. Introduction

Fabry disease (FD) is a X-linked recessive lysosomal storage disorder caused by the total or partial deficiency of the lysosomal hydrolase α-galactosidase A enzyme (α-gal A), involved in the biodegradation of glycosphingolipids, resulting in a progressive deposition of non-catabolized metabolites within the lysosomes of different cells and organs [1].

The prevalence of this disease is estimated to be between 0.85 and 2.5 cases per 100,000 individuals worldwide, but it may be largely underestimated due to the wide spectrum of clinical phenotypes [2]. Indeed, FD is a multisystem pathology, involving renal, cardiovascular, neurological, dermatological, and ophthalmological systems [3], that, if untreated, leads to severe complications that may end in premature death [4,5]. For these reasons, the identification of early biomarkers is crucial in this condition. In this light, it is noteworthy to mention that ophthalmologic signs are some of the earliest manifestations of this disease, and could therefore represent important markers for the diagnosis [6].

The most common ocular signs are cornea verticillata, lens opacities, and conjunctival and retinal vessel tortuosity, along with aneurysmal dilatations [7,8,9]. In particular, retinal vessel abnormalities, probably secondary to glycosphingolipid deposits within the vessel walls (especially in smooth muscle and endothelial cells), result in modification of their structure and resistance to hydrostatic pressure [10,11]. These vascular abnormalities can be detected by fundus examination, which however provides a subjective and poorly reproducible evaluation [6].

In this light, optical coherence tomography angiography (OCT-A) allows for an objective analysis of retinal microvasculature alterations [12]. In fact previous studies showed significant changes in retinal vascular networks using OCT-A [13,14,15]. The purpose of this study was to carry out, using OCT-A, a quantitative analysis of the superficial and deep capillary plexus (SCP and DCP, respectively) in a large sample of patients with FD, with the aim of further expanding the knowledge about ocular alterations in this condition.

## 2. Experimental Section

### 2.1. Subjects

A total of 108 eyes from 54 FD patients (34 females, 20 males, mean age 44.1 ± 15.6 years) were enrolled from July 2017 to May 2018 in the Eye Clinic of the University of Naples “Federico II”. 

The diagnosis of classic FD was defined by typical clinical characteristics of the disease, family history, and reduced plasma levels of α-galactosidase A enzyme activity to less than average normal values, with confirmation by genotyping tests. All patients proved to have a pathogenetic mutation, and no subjects with late onset or variants of unknown significance were included in this study.

For all patients, signs of cardiac, renal, and central nervous system (CNS) involvement were recorded from clinical records and magnetic resonance imaging (MRI) scans. In particular, according to previous reports [16,17,18], cardiac involvement was declared positive if arrhythmia or left ventricular hypertrophy were present, while renal failure was defined if an estimated glomerular filtration rate <90 mL/min was present. Finally, CNS involvement was defined if MRI signs of the disease were present, as follows. For each patient for whom an MRI scan was available (41/54), images were examined in consensus by two neuroradiologists (GP and CR) to assess the presence of cerebral macrovascular events (namely stroke) [19], while the severity of microvascular disease was determined according to the total MRI brain small vessel disease (SVD) score [20]. Patients with MRI signs of stroke or a total SVD score ≥1 were considered affected.

Along with FD patients, 70 healthy subjects (HS) (36 females, 34 males, mean age 42.3 ± 15.6 years) with a normal ophthalmic examination and no history of intraocular surgery or retinal pathologic features were included as control group.

Each subject underwent an evaluation of best corrected visual acuity (BCVA) according to the Early Treatment of Diabetic Retinopathy Study (ETDRS), intraocular pressure measurement, slit-lamp biomicroscopy, fundus examination with a +90 D lens and indirect ophthalmoscopy, multicolor images, spectral domain (SD)-OCT (Spectralis + HRA; Heidelberg Engineering, Heidelberg, Germany), and OCT-A (RTVue XR Avanti, Optovue, Inc., Freemont, CA, USA).

Exclusion criteria were clinically relevant opacities of the optic media, cornea verticillata, low-quality OCT-A images, myopia greater than 6 diopters, history of intraocular surgery, evidence of vitreoretinal and macular disease, uveitis, diabetic retinopathy, congenital eye disorder, and other ocular pathologic features (e.g., combined retinal vein and artery occlusive disease).

The study was approved by the Institutional Review Board of the University of Naples “Federico II” and all investigations adhered to the tenets of the Declaration of Helsinki. Written informed consents were obtained from the patients enrolled in the study.

### 2.2. OCT-A

OCT images were acquired with the Angiovue System (RTVue XR Avanti, Optovue, Inc., Freemont, CA, USA), which is based on split-spectrum amplitude de-correlation system (SSADA). The instrument has an A-scan rate of 70,000 scans per second with a tissue axial resolution of 5 µm and a 15 µm beam width. Each B-scan contained 304 A-scans. Two consecutive B-scans were captured at a fixed position before proceeding to the next sampling location. Size volumes were recorded and the B-scan images were compared with each other to calculate decorrelation in the images. Blood flowing through vessels causes a change in reflectance over time and results in localized areas of flow de-correlation between frames. The spectrum of the light source was split into multiple component parts to decrease the noise present in the image; each part was used to perform the de-correlation step and the results of all the split spectra were averaged. In any given region of tissue, the projection image can be viewed to obtain an image of the contained blood flow [21]. The projection artifact removal software was used in order to ensure correct visualization of the SCP and DCP.

Cross-sectional registered reflectance intensity images and flow images were summarized and viewed as an en face maximum flow projection from the inner limiting layer to the retinal epithelial pigment. The macular capillary network was visualized in scans centered on the fovea by performing a 6×6 mm scan over the macular region. Vessel density (VD) was defined as the percentage area occupied by the large vessels and microvasculature in the analyzed region [22].

The OCT-A software, according to the ETDRS classification of diabetic retinopathy, analyzed the macular region divided in whole image, fovea, and parafovea. For each eye analyzed, the software (AngioAnalytic™) automatically calculated vessel density in different vascular networks of the retina: the SCP and DCP.

Poor-quality images with a signal strength index (that reflects OCT image quality) of less than 40 or registered image sets with residual motion artefacts were excluded from the analysis. 

### 2.3. Statistical Analysis

Statistical analysis was performed with the Statistical Package for Social Sciences (Version 17.0 for Windows; SPSS Inc., Chicago, IL, USA).

Student’s *t*-test and chi-squared test were used to determine between group differences in terms of age and sex, respectively, with a *p*-value < 0.05 considered statistically significant. 

Form the enrolled subjects, one eye was randomly selected for the subsequent between-group comparisons analysis, leaving a total number of 54 and 70 eyes for FD and HS, respectively. To evaluate differences in VD in each capillary plexus between controls and patients, a general linear model (GLM) was performed, including age and sex as confounding covariates, with a significance level set for *p <* 0.008, and Bonferroni corrected for multiple comparisons (as 0.05/6, the number of tested OCT-A variables) 

## 3. Results

The two groups were not significantly different for age (44.1 ± 15.6 and 42.3 ± 15.6 for FD and HS, respectively; *p =* 0.62) and sex (male/female= 20/34 and 29/31 for FD and HS, respectively; *p =* 0.30).

Among the patients, 43/54 (79.6%) showed presence of cardiac involvement, 8/54 (14.8%) renal involvement, 7/41 (17.1%) neuroradiological signs of CNS involvement, and 38/54 (70.4%) subjects were under enzyme replacement therapy at the moment of the examination. 

The demographic and clinical information of controls and FD patients are listed in Table 1. 

Eighteen FD subjects (35.2%, M/F = 4/14) showed the presence of cornea verticillata, while in seven patients (13.0%, M/F = 4/3) lenticular opacities were present. Visual acuity examination showed no statistically significant difference in BCVA between controls and patients (0.02 ± 0.04 logMar vs. 0.03 ± 0.09 logMar; *p* = 0.29).

The ophthalmologic characteristics of FD patients are provided in Table 2.

Compared to HS, FD patients showed lower VD values in the SCP in the whole image (49.95 ± 5.17% vs. 51.99 ± 2.52%; *p* < 0.001), as well as in the parafovea (52.01 ± 6.69% vs. 54.30 ± 2.61%; *p* = 0.002), and fovea (22.38 ± 9.01% vs. 29.31 ± 5.84%; *p* < 0.0001). On the other hand, in the DCP VD values were significantly higher in patients compared to controls in the whole image (54.82 ± 8.07% vs. 50.93 ± 5.46%; *p* = 0.005), as well as in the parafovea (57.76 ± 7.26% vs. 53.59 ± 5.46%; *p* = 0.0001), and fovea (39.75 ± 8.59% vs. 34.43 ± 8.68%; *p* < 0.0001). 

**Figure 1:**
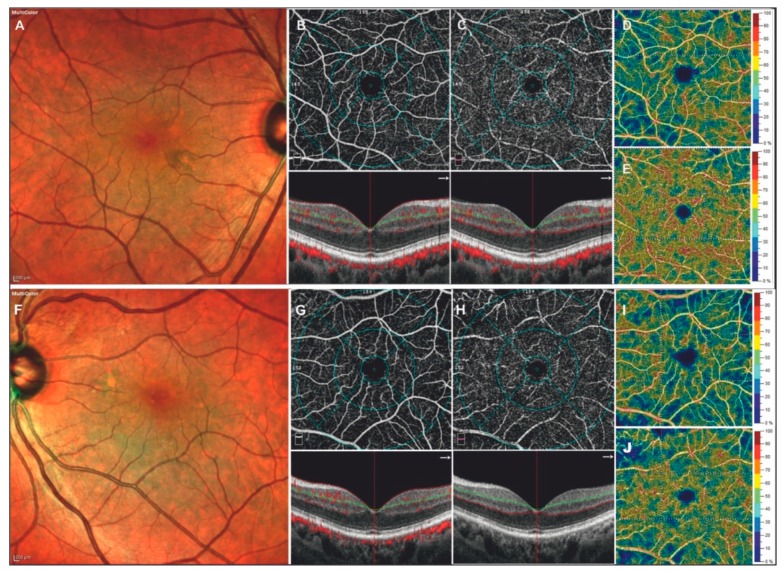
Right and Left eyes of a 68-year-old patient with Fabry disease. Multicolor image shows a qualitative increase of retinal vascular tortuosity (**A**,**F**). Optical coherence tomography angiography and vessel density map showed a reduction in the superficial capillary plexus (**B**,**D**); (**G**,**I**), coupled to an increase in the deep capillary plexus (**C**,**E**); (**H**,**J**).

A complete list of the between group analysis regarding VD values is available in Table 3, while an example of the OCT-A analysis is shown in Figure 1.

## 4. Discussion

In this prospective study we evaluated, using OCT-A, a quantitative analysis of retinal VD in macular region in FD patients. We found a complex and heterogeneous involvement of VD in this condition, with a different behavior between the SCP and DCP. In particular, we found in the SCP a lower VD in patients compared to controls, while an increase in VD at the level of the DCP was found in our study population.

A possible explanation to this different behavior could be researched, in first hypothesis, in the remodeling of the vessel walls secondary to the Gb3 accumulation in smooth muscle and vascular endothelial cells [10]. This accumulation, which determines vascular narrowing and tortuosity [23,24,25], could lead to a reduction of the blood flow, which may subsequently be reflected as a lower VD in the SCP, evaluated by OCT-A.

On the other hand, reduction of the VD at the level of the SCP could also be due to a state of abnormal blood flow and hypercoagulability reported in FD [25]. Indeed, a study conducted by DeGraba et al. [26] found significantly higher levels of platelet adhesion and aggregation factors in plasma of patients of FD patients, possibly predisposing them to retinal vascular occlusions [23,27,28].

Independent of the physiopathological mechanism behind the VD reduction in the SCP of FD patients, we speculate that changes affecting this area could be an early phenomenon related to the other major finding of the present work. Indeed, when analyzing alterations of the DCP, we found a significant increase in the VD in patients compared to controls. This result, although counterintuitive, could be interpreted as a compensatory vascular mechanism to support the abovementioned reduction of VD in the SCP. 

Indeed, the deep vascular network is known to be characterized by a large number of small capillaries, connected to the SCP through multiple vertical anastomoses [12].

It is therefore possible to hypothesize that the increased recruitment of microvascular units in DCP, in response to the reduction of VD in the SCP, could lead to an increase in hydrostatic pressure at deeper levels. This change in the hydrostatic pressure could lead to marked capillary congestion and vascular dilatation, and also to the weakening of their resistance to transmural pressure [11]. In line with this speculation, in other retino-vascular disorders characterized by vessel tortuosity and dilatation, such as retinal vein occlusion, vascular congestion was mainly observed in the DCP [29,30].

Previous studies, using retinography and fluorescein angiography, have performed descriptive analyses of the posterior segment in FD patients showing an increase in retinal vascular tortuosity and dilatation [6]. Nevertheless, these tools provide a subjective and poorly reproducible evaluation, and for these reasons, a more quantitative and objective diagnostic analysis might be useful in the evaluation of FD patients. 

Indeed, previous studies have shown how computer-assisted systems were capable of measuring the retinal vessel tortuosity in FD [6,31], with the parameters that were higher in patients compared with controls being associated with clinical scores. 

Using OCT-A, Hufendiek et al. showed a significant reduction in the SCP and DCP compared to controls (37.9% vs. 47%; 31.9% vs. 41.6%, respectively) while Baur and Finocchio revealed a significant reduction only in DCP (0.47 vs. 0.49; 3.05 mm² vs. 3.32 mm², respectively) [13,14,15].

Nevertheless, different limitations should be acknowledged in the present study. In particular, the main limitation of this work relies in its cross-sectional nature, which limits all the hypotheses about the different behavior between SCP and DCP to speculative, with particular reference to the possible early involvement of the SCP and its relation to the development of DCP abnormalities. For this reason, future longitudinal studies are warranted to further confirm or dismiss this hypothesis. Another limitation of the present study is the absence of other specific vascular markers, such as the evaluation of the diameters of the brain’s posterior circulation, which have been reported as increased in FD patients [32]. Future studies correlating different imaging biomarkers are therefore warranted to evaluate a possible relation between these changes.

Although these limitations are present, in conclusion our study for the first time provides evidence about a complex pattern of microvascular changes affecting the retinal vascular network of FD patients. Given that this condition is characterized by significant systemic vascular involvement, a detailed evaluation of retinal vascular perfusion, using an objective and reproducible tool such as OCT-A, may contribute to early diagnosis.

## Figures and Tables

**Table 1:** Demographic and systemic information in controls and in patients with Fabry disease.

	Controls	Patients
Patients *n*.	70	54
Sex (male/female)	29/31	20/34
Age (years; mean ± SD)	42.3 ± 15.6	44.1 ± 15.6
Cardiac involvent	-	43/54 (79.6%)
Renal failure	-	8/54 (14.8%)
Neurological involvement	-	7/41 (17.1%)
ERT	-	38/54 (70.4%)

SD: standard deviation; ERT: enzyme replacement therapy.

**Table 2:** Ophthalmologic characteristics in controls and patients with Fabry disease.

	Patients
Number of eyes	108
Cornea verticillata (Male/Female)	4/14
Lenticular opacities (Male/Female)	4/3
BCVA, logMAR (Snellen)	0.03 ± 0.09 (20/20)

BCVA: best-corrected visual acuity; logMAR: logarithm of the minimum angle of resolution (Snellen equivalent in brackets).

**Table 3:** Differences in Optical coherence tomography angiography vessel density between controls and patients with Fabry disease.

	Controls	Patients	*p*-Value
Superficial capillary plexus (%)			
Whole image	51.99 ± 2.52	49.95 ± 5.117	<0.001
Parafovea	54.30 ± 2.61	52.01 ± 6.69	0.002
Fovea	29.31 ± 5.84	22.38 ± 9.01	<0.0001
Deep capillary plexus (%)			
Whole image	50.93 ± 5.46	54.82 ± 8.07	0.005
Parafovea	53.59 ± 5.46	57.76 ± 7.26	0.0001
Fovea	34.43 ± 8.68	39.75 ± 8.59	<0.0001

Data expressed as mean ± SD. General linear model, *p* < 0.008.
